# Developmental Venous Anomaly Presenting with Spontaneous Intracerebral Hemorrhage, Acute Ischemic Stroke, and Seizure

**DOI:** 10.7759/cureus.5412

**Published:** 2019-08-17

**Authors:** Naresh Mullaguri, Anusha Battineni, Balaji Krishnaiah, Ibrahim Migdady, Christopher R Newey

**Affiliations:** 1 Neurology, Cleveland Clinic Foundation, Cleveland, USA; 2 Vascular Neurology, University of Tennesse, Memphis, USA

**Keywords:** cavernous malformation, intracerebral hemorrhage (ich), developmental venous anomaly, ischemic stroke, seizures, venous hypertension

## Abstract

Developmental venous anomaly (DVA) is the most common, benign, congenital vascular malformation of the brain and mostly an incidental finding on imaging. The exact etiology of DVA is unknown but thought to be due to medullary vein thrombosis during embryonic venous development. DVA is generally asymptomatic although associated neurologic deficits and seizures have been described. Several reports of DVA causing neurovascular compression, obstructive hydrocephalus, venous infarction, and intracerebral hemorrhage (ICH) have been described. In this report, we discuss a patient with fluctuating neurological symptoms found to have multiple DVA, predominantly draining into the deep venous system. To the best of our knowledge, DVAs leading to simultaneous ischemic stroke, intracerebral hemorrhage, and seizures are not reported in the literature. We reviewed the relevant literature and discussed the epidemiology and clinical and radiological characteristics of DVA.

## Introduction

Developmental venous anomaly (DVA) is the most common congenital vascular malformation of the brain. Its prevalence is 2.6% of all vascular malformations in a large autopsy series [1]. It is mostly an incidental finding in neuroimaging and considered benign. The detection rate is 0.48%-0.7% after the advent of magnetic resonance imaging (MRI) [2]. DVA mostly drains the normal brain parenchyma. Occasionally, it may serve as a draining channel for dural arteriovenous (AV) fistulas and AV malformations [3]. Structurally, DVA constitutes several small medullary veins draining into a large collecting vein simulating the ‘caput medusa' appearance. DVA is classified as a superficial or deep type based on the drainage of collecting vein. A superficial type DVA drains into cortical veins and dural venous sinuses. Deep type DVA flows predominantly into the Galenic system. Cavernous malformations (CM) can develop in the DVA draining territory due to altered cerebral hemodynamics [4-5]. Approximately 10%-30% of DVA patients harbor CM and their prevalence increases with age [4]. DVA coexisting with CM has a higher incidence of intracerebral hemorrhage, focal epilepsy, headache, and other focal neurological deficits. It is thought to be secondary to CM rather than DVA [2]. DVAs complicating neurovascular compression, obstructive hydrocephalus, venous infarction, and intracerebral hemorrhage (ICH) is well known. They are called idiopathic symptomatic DVAs [3]. To the best of our knowledge, no cases of DVAs presenting with simultaneous acute ischemic stroke, intracerebral hemorrhage, and recurrent seizures were described in the literature so far.

## Case presentation

**Figure 1:**
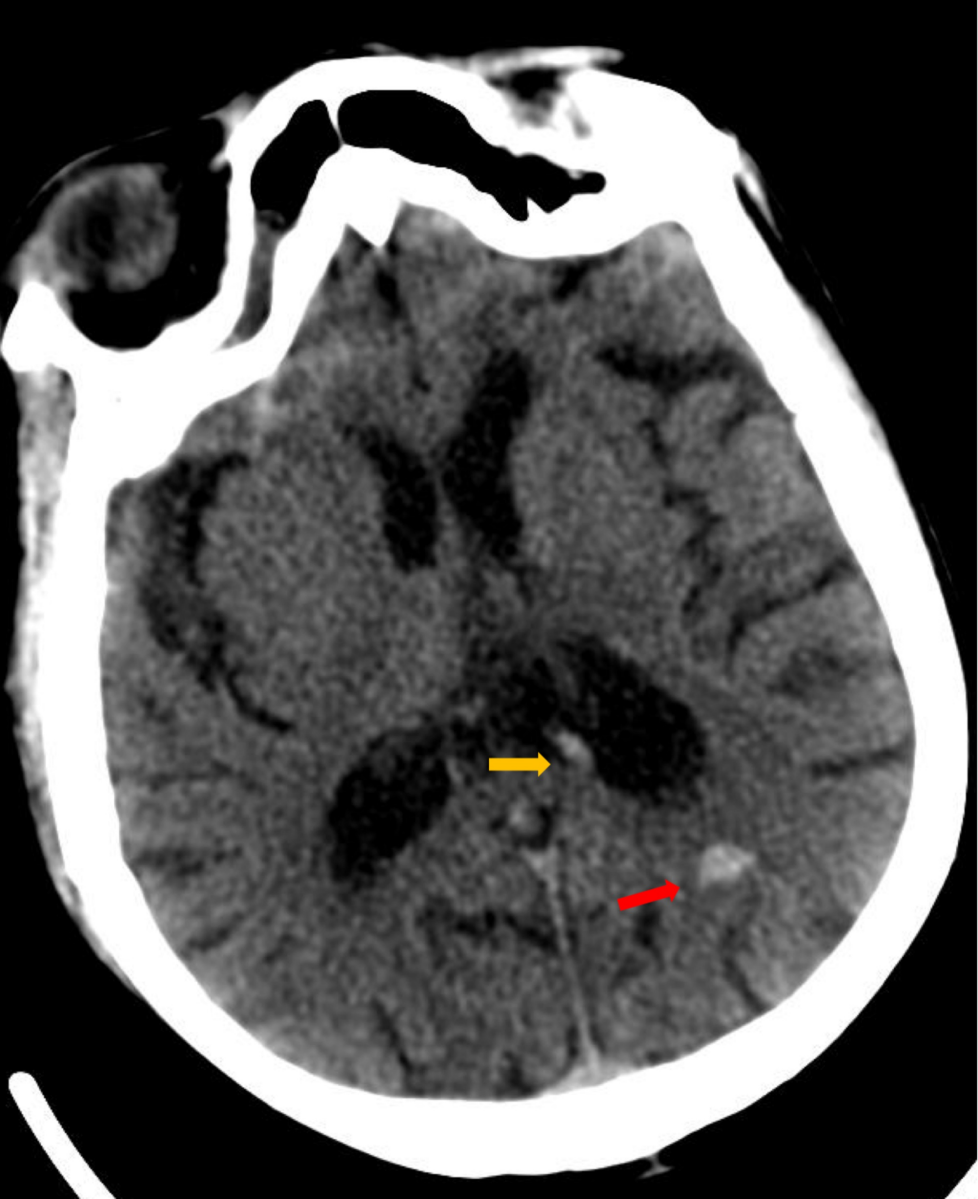
Computerized tomography of the brain Axial section showing a small left parietal lobe subcortical hemorrhage (red arrow) with a hyperdense ependymal vein (yellow arrow)

A 65-year-old African-American male with a history of hypertension, hyperlipidemia, and alcohol abuse presented with acute-onset right-sided weakness, gaze preference, and speech difficulties. Family members denied any falls, headache, fever, seizures, prior history of stroke, excessive alcohol consumption, or usage of recreational drugs lately. He was evaluated in the mobile stroke treatment unit by a neurologist with a concern for acute stroke. His vital signs were remarkable for sinus tachycardia of 104 beats per minute and blood pressure of 132/82 mm Hg. His neurological examination showed right-sided hemiparesis and aphasia with hyperreflexia in all the extremities. Initial National Institutes of Health Stroke Scale (NIHSS) was 14 and suspicious for left middle cerebral artery syndrome. His blood work is remarkable for leukocytosis of 13,000 k/uL (normal range 3700-1,700/uL). A non-contrast computerized tomography (CT) scan of the head showed intracerebral hemorrhage (ICH) in the left parietal lobe. It also showed a large sub-ependymal hyperdense vessel next to the hemorrhage suspicious for a cavernous malformation with a draining vein (Figure 1).

**Figure 2:**
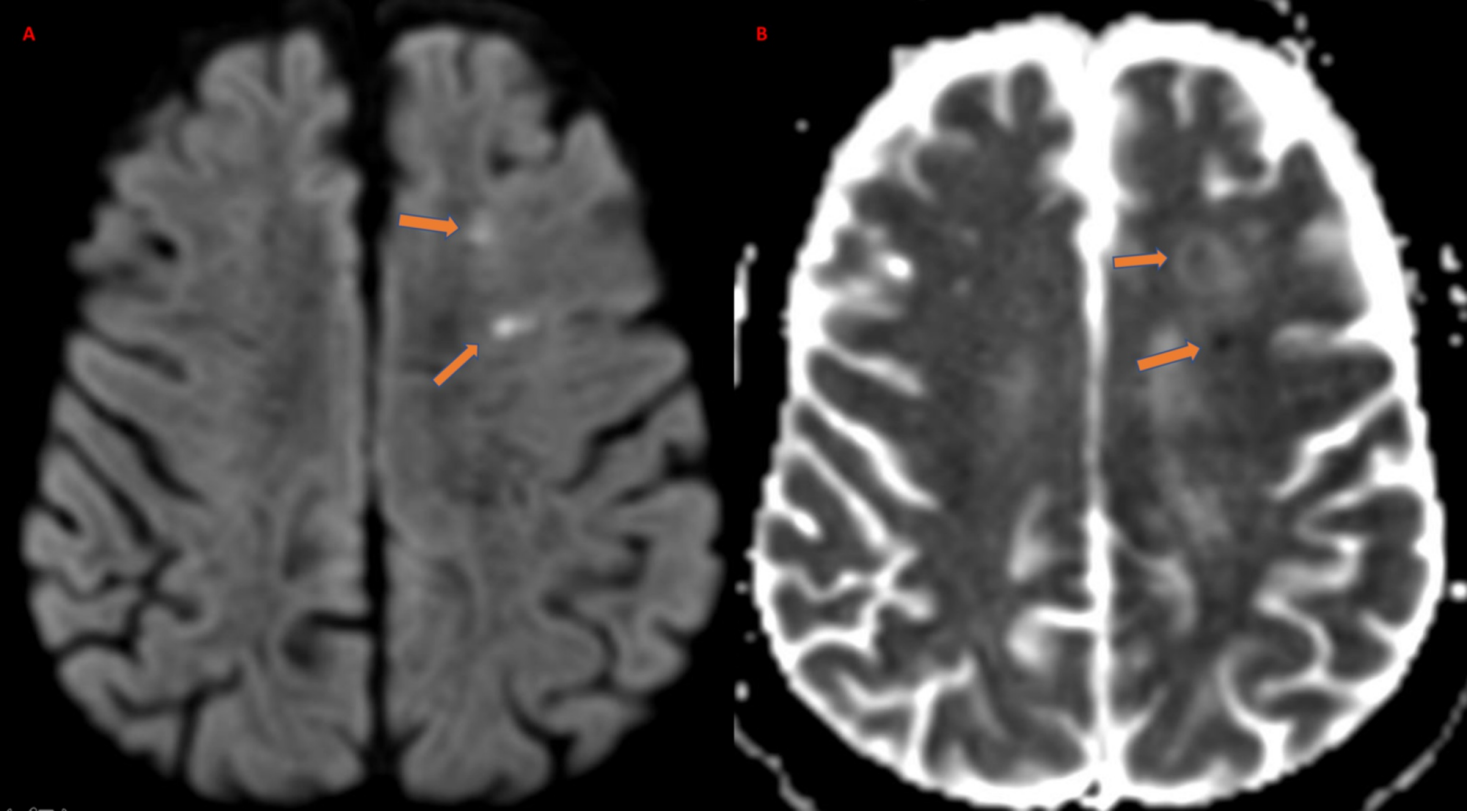
Magnetic resonance imaging of the brain Image A - Diffusion-weighted magnetic resonance imaging (DWI) sequence showing acute infarction in the left frontal subcortical region (orange arrows). Image B - the corresponding apparent diffusion coefficient (ADC) image showing diffusion restriction (orange arrows).

His weakness improved rapidly to an NIHSS of 4 with residual confusion and aphasia. CT angiogram of the head did not show large vessel occlusion or vascular malformations. Given the clinical-neuroimaging mismatch, a focal seizure secondary to ICH was considered a possibility in addition to ischemic stroke. Hypertension, cerebral amyloid angiopathy, and venous sinus thrombosis were considered as the differential diagnosis of ICH. Laboratory testing showed an unremarkable urine toxicology screen. The glycated hemoglobin was 5.2% (normal range 4.3%-5.6%) and low-density lipoprotein was elevated at 135 mg/dL (normal range 60-129 mg/dL). MRI of the brain without contrast showed punctate areas of diffusion restriction in the left frontal periventricular region (Figure 2).

**Figure 3:**
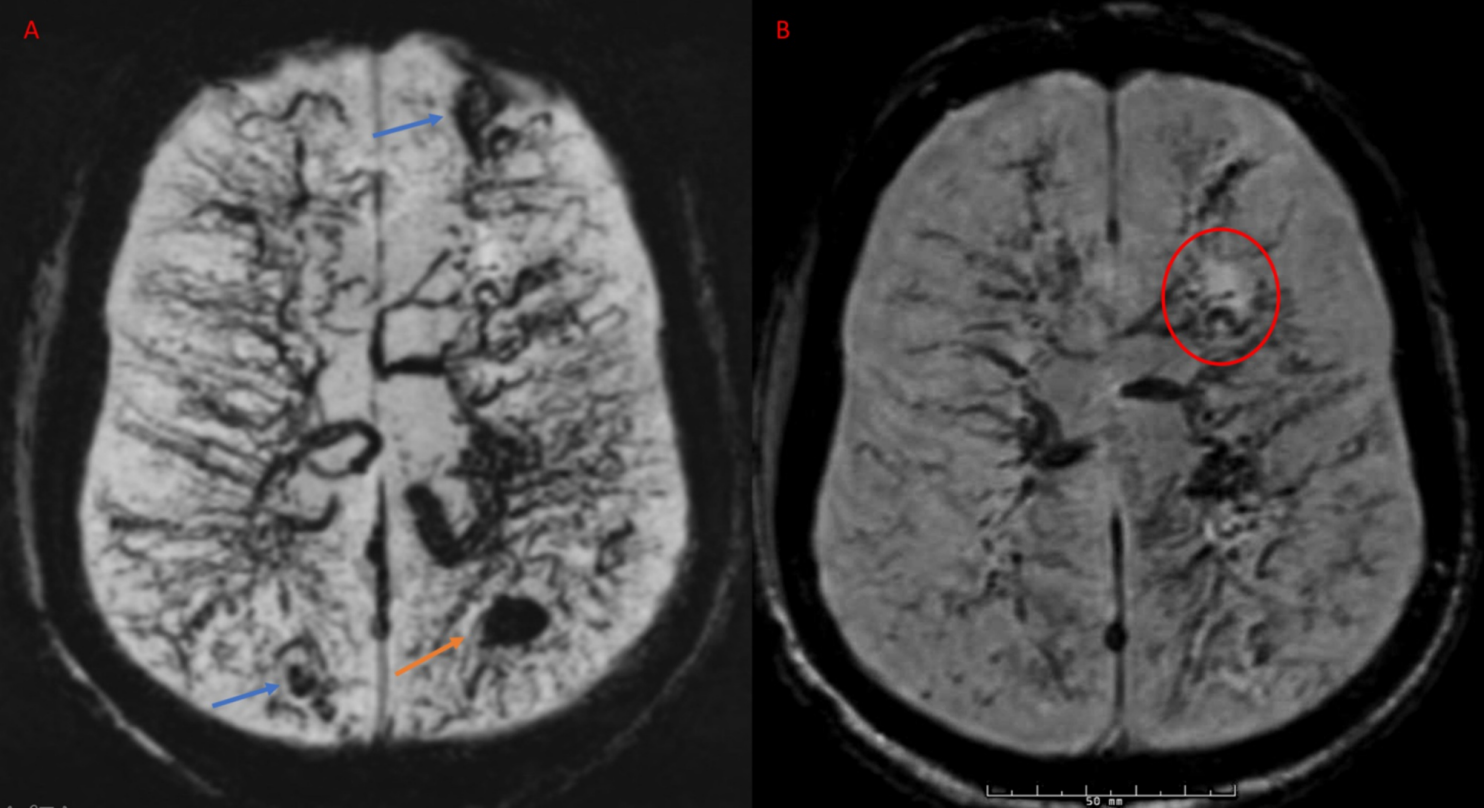
Magnetic resonance susceptibility-weighted imaging (SWI) axial sections Images A and B showing multiple DVAs with serpentine deep veins and the ‘caput medusa’ appearance. There are multiple old hemorrhages in the right parietal and left frontal areas (blue arrows) with acute left parietal hemorrhage (orange arrow). A ‘halo’ sign is noticed in the left frontal area around the medullary veins in the area of infarction in image B (red circle).

Susceptibility-weighted imaging (SWI) showed multiple developmental venous anomalies (DVAs) in bilateral cerebral and cerebellar hemispheres simulating the ‘caput medusa’ appearance. Several old microhemorrhages in the anterior and posterior circulation (not shown) and stable left parietal acute ICH are seen (Figure 3).

**Figure 4:**
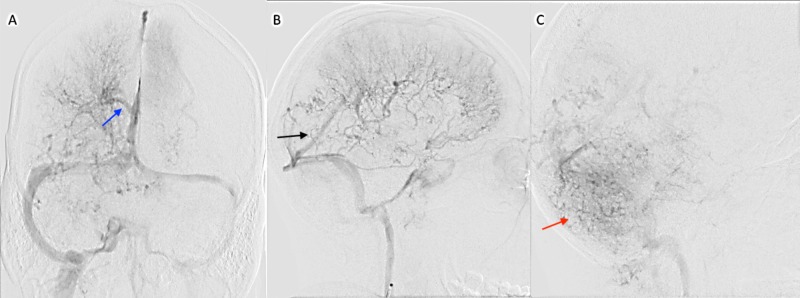
Digital subtraction angiography of the brain venous phase showing venous congestion in the bilateral cerebral and cerebellar hemisphere. Image A – right internal carotid artery injection posterior-anterior view showing developmental venous anomaly draining into the straight sinus (blue arrow). Image B – lateral view of the left cerebral hemisphere showing the absence of cortical veins with predominant venous drainage into the subependymal veins. Due to the underdevelopment of the superior sagittal sinus, the majority of veins drain into deep venous system (black arrow). Image C – vertebral artery injection showing severe venous congestion in the cerebellar hemisphere (red arrow).

MRI cervical spine to evaluate diffuse hyperreflexia showed chronic spinal cord compression at the C3-C4 and C4-C5 segments. Neurosurgery did not recommend any surgical intervention. Digital subtraction angiography (DSA) showed diffuse subcortical venous congestion with serpentine medullary veins in the bilateral cerebral and cerebellar hemispheres. They drain predominantly into the vein of Galen and straight sinus. There is an absence of cortical venous drainage with underdeveloped superior sagittal sinus. No evidence of dural or cortical venous thrombosis or arteriovenous shunting was present (Figure 4).

His hemiparesis and aphasia worsened immediately after the angiogram, and his blood pressure was 184/94 mmHg. He was given intravenous boluses of labetalol and hydralazine. His symptoms improved in a couple of hours while the systolic blood pressures were in the range of 130-140 mmHg. He continued to fluctuate during the hospitalization with systolic blood pressures of more than 160 mmHg. Electroencephalography (EEG) monitoring didn’t show any seizures or epileptiform discharges during these spells. It showed cortical dysfunction maximum in the left central-temporal region with mild diffuse encephalopathy. Reversible encephalopathy causes, including thyroid profile and infectious and metabolic workup, were unremarkable. Repeat CT scans of the brain during these episodes showed no new changes. He was started on amlodipine 10 mg, which brought his systolic blood pressures down to the range of 110-150 mmHg with no further episodes. He was started on aspirin and atorvastatin for secondary prevention of ischemic stroke. He was discharged to acute rehabilitation with an NIHSS of 8 for right-sided weakness and aphasia. His discharge modified Rankin score was three (range 0-6; 0 means no symptoms and 6 means death).

On Day 10 at the rehabilitation facility, he had an episode of right-sided upper and lower extremity rhythmic shaking. It progressed to a generalized convulsion lasting a minute with post-ictal confusion. He was readmitted to the hospital with an admission NIHSS of 15 for an impaired level of consciousness, right-sided weakness, and aphasia. Repeat CT scan of the brain did not show any new findings and stable intracerebral hemorrhage. He was diagnosed with complex partial seizures secondary to a recent stroke or hemispheric DVA with venous congestion. He was started on oral levetiracetam for seizure prevention but switched to zonisamide due to agitation. EEG monitoring for 48 hours showed no seizure activity and was discontinued. He had another breakthrough complex partial seizure the following day, and his zonisamide dose was increased. EEG monitoring for the subsequent 48 hours did not show clinical/electrographic seizures other than diffuse encephalopathy. He was discharged to the skilled nursing facility on Day 16. His discharge NIHSS was 15 for the level of consciousness questions, commands, right-sided weakness, and aphasia. His discharge modified Rankin score was 4. He was lost to follow-up.

## Discussion

Etiopathogenesis and epidemiology

DVA is the most common venous malformation of the cerebrovascular system, constituting about 60% of all vascular malformations and is mostly incidental on neuroimaging [3]. The exact prevalence is unknown, but autopsy studies have reported an incidence of 2%-3% in the general population [1]. Up to 80% are supratentorial areas, with the remaining 20% in the cerebellar hemispheres and brainstem [3]. The exact etiology of DVA is unknown but thought to be due to medullary vein thrombosis during embryonic venous development, resulting in the persistence of primordial venous drainage patterns. Histologically, they appear as thin, fragile veins inter-spread in the brain parenchyma draining into a single collecting vein, which is devoid of tunica media [6]. Chronic venous hypertension around DVA is thought to cause changes of demyelination, leukomalacia, and gliosis in the brain parenchyma.

Pathomechanisms and clinical features

Pereira et al. classified the pathomechanisms of DVA into (i) mechanical, when the dilated collecting vein obstructs the natural cerebrospinal fluid (CSF) pathways mostly in the aqueduct and fourth ventricle; (ii) flow-related, with increased outflow causing venous hypertension, resulting in hemorrhagic complications or decreased outflow from the anatomical obstruction of the collecting vein leading thrombosis; and (iii) idiopathic symptomatic DVAs with no known associated findings to explain the complications [3]. He also reported 38% of patients developed focal neurological deficits (FND) in DVA patients with increased outflow and in 69% of DVAs with outflow restriction [3]. The fluctuating neurological symptoms in our patient are time-locked to elevated blood pressure, which might have increased venous congestion, leading to clinical worsening. The improvement in his symptoms with systolic blood pressures under 160 mmHg suggests compensated venous outflow with reduced congestion.

Seizures complicate 1%-4% of patients with DVAs by various mechanisms, including CMs, stroke, abnormal neuronal migration during embryonic development, and focal cortical dysplasia [2-3]. EEG monitoring in our patient showed severe diffuse encephalopathy with slowing predominantly in the left hemisphere suggestive of decreased cerebral blood flow due to restricted venous outflow [7]. The episodes of worsening right-sided weakness during the initial hospitalization with no epileptiform discharges in EEG might be due to progressive ischemia and hypometabolism from venous congestion [8-9].

Developmental venous anomaly and stroke

Intracerebral hemorrhage is a rare complication of DVAs and mostly described in patients with coexisting vascular malformations. DVAs can be symptomatic due to altered hemodynamics resulting in venous hypertension and congestion causing spontaneous and recurrent ICH, which we think happened in our patient [10-12]. Cerebral angiography did not reveal any associated vascular malformations, stenosis, or thrombosis of the collecting vein, instead showed multiple supra and infratentorial DVAs with diffuse subcortical venous congestion. McLaughlin et al., in their observational study of 80 patients with symptomatic DVAs, found an annual hemorrhage rate of 0.68% per year retrospectively and 0.34% per year in the prospective group [10]. Garner et al. found an annual hemorrhage risk of 0.22% [11]. These hemorrhages are usually small and do not lead to increased mortality [10]. Up to 30% of patients with DVAs have associated CMs and rarely dural arteriovenous fistula (DAVF) or dural arteriovenous fistulas (AVMs) [13]. Chronic venous hypertension in the draining region of DVAs promotes the formation of CMs due to erythrocyte diapedesis and growth factor release [14]. Pereira et al. reported an increased incidence of hemorrhagic complications with posterior circulation DVAs using deep venous drainage and correlates in our case as evidenced by bilateral cerebellar microhemorrhages, but the exact mechanism is unknown [3]. An MRI SWI sequence of the brain showed multiple old macro and microhemorrhages, mostly in the left frontal and bilateral cerebellar hemispheres, in addition to the acute left parietal hemorrhage. Hypertension and cerebral amyloid angiopathy (CAA) are the most common causes of recurrent ICH. In our patient, multiple DVAs either by forming CMs or due to venous hypertension in their draining territory is hypothesized to have caused recurrent ICH.

Symptomatic DVA complicating cerebral infarction is rare and constitute 1% of patients with DVA [3,10]. We hypothesize that the left frontal deep white matter draining DVA is affected by decreased venous outflow and congestion as seen in cerebral angiography, resulting in ischemia and infarction. Given the exclusive deep venous drainage for the whole left hemisphere, we hypothesize that the left parietal DVA draining into the sub-ependymal vein is overloaded to drain most of the left hemisphere in the absence of cortical venous drainage, resulting in venous hypertension and hemorrhage in its territory. These multiple deep type DVAs may have dynamic variations in their outflow resulting in venous congestion and hypertension at the same time causing simultaneous infarction and hemorrhage. Chronic venous hypertension can cause changes in the collecting veins, resulting in the thickening of the walls and reduced drainage as evidenced by the incidence of stroke symptoms between the fourth and sixth decades of life [15-16]. Most DVAs are not amenable to surgical management, as they drain the normal brain parenchyma and doing so results in a risk of venous infarction [10]. Given these extreme venous drainage patterns with no associated vascular malformations complicating the DVAs, he was managed medically with antiepileptic medications and aspirin.

Neuroimaging characteristics

MRI of the brain with T1 post-contrast and SWI demonstrate the 'Medusa head' appearance and is considered the diagnostic modality of choice for the diagnosis of DVAs [13,17]. Cerebral angiography is also used to identify venous congestion, drainage patterns, occlusion, or thrombosis of the collecting vein and associated vascular malformations [18]. CT, MRI, and perfusion-weighted imaging (PWI) studies showed increased relative mean transit time (MTT), relative cerebral blood volume (CBV), and varied relative cerebral blood flow (CBF) in the drainage area of DVAs [5,19]. Functional studies, such as single positron emission computerized tomography (SPECT), may demonstrate hypometabolism around the DVAs. These findings are suggestive of decreased outflow and venous congestion [8-9].

## Conclusions

A thorough angiographic evaluation is warranted to find associated vascular malformations in patients with DVAs. Complex hemispheric DVAs can become symptomatic with advancing age, possibly due to changes in the medullary and collecting veins secondary to long-standing venous hypertension. DVA causing simultaneous ischemic and hemorrhagic stroke is rare and must be considered in the differential diagnosis. Fluctuating neurological symptoms in patients with DVA can be due to increased venous congestion causing low cerebral blood flow or recurrent seizures.
